# Assessment of Seroconversion after SARS-CoV-2 Vaccination in Patients with Lung Cancer

**DOI:** 10.3390/vaccines10040618

**Published:** 2022-04-15

**Authors:** Ioannis P. Trontzas, Ioannis Vathiotis, Christina Economidou, Ioulia Petridou, Georgia Gomatou, Maria Grammoustianou, Ioannis Tsamis, Nikolaos Syrigos, Maximilian Anagnostakis, Eleni Fyta, Vissaria Sakka, Garyphalia Poulakou, Elias A. Kotteas, Ekaterini Syrigou

**Affiliations:** 1 Oncology Unit, 3rd Department of Internal Medicine, “SOTIRIA” General Hospital of Chest Diseases, National and Kapodistrian University of Athens, 11527 Athens, Greece; johnvathiotis1@gmail.com (I.V.); georgiagom@med.uoa.gr (G.G.); marygrm90@gmail.com (M.G.); dr_tsamis_ioannis@hotmail.com (I.T.); nsyrigos@hsph.harvard.edu (N.S.); maximilian2002@gmail.com (M.A.); efyta@med.uoa.gr (E.F.); ilkotteas@med.uoa.gr (E.A.K.); 2 Affidea Diagnostic Center, 17675 Athens, Greece; christina.economidou@affidea.com (C.E.); ioulia.petridou@affidea.com (I.P.); 3 Department of Epidemiology, Harvard T.H Chan School of Public Health, Boston, MA 02115, USA; 4 Unit of Infectious Diseases, 3rd Department of Internal Medicine, “SOTIRIA” General Hospital of Chest Diseases, National and Kapodistrian University of Athens, 11527 Athens, Greece; vissaria@gmail.com (V.S.); gpoulakou@med.uoa.gr (G.P.); 5 Allergy Department, “SOTIRIA” General Hospital of Chest Diseases, 11527 Athens, Greece; laboratest@yahoo.gr

**Keywords:** COVID-19, SARS-CoV-2, lung cancer, vaccination, seroconversion, humoral immunity, antibodies

## Abstract

*Background*: SARS-CoV-2 mortality rates are significantly higher in patients with lung cancer compared with the general population. However, little is known on their immunization status after vaccination. *Methods*: To evaluate the humoral response (seroconversion) of patients with lung cancer following vaccination against SARS-COV-2 (Group A), we obtained antibodies against SARS-CoV-2 spike (S) protein both at baseline and at different time points after the first dose of SARS-CoV-2 vaccine (two to three weeks [T1], six weeks ± one week [T2], 12 weeks ± three weeks [T3], and 24 weeks ± three weeks [T4]). Antibodies were also acquired from a control cohort of non-lung cancer patients (Group B) as well as a third cohort containing healthy controls (Group C) at all time points and at T4, respectively, to make comparisons with Group A. Analysis of antibody response at different time points, association with clinicopathologic parameters, and comparisons with control groups were performed. *Results*: A total of 125 patients with lung cancer were included in the analysis (96 males [74.3%], median age of 68 years [46–91]. All study participants received two vaccine doses (BNT162b2, mRNA-1273, AZD1222). Analysis of anti-SARS-CoV-2 S antibody titers showed minimal response at T1 (0.4 [0.4–48.6] IU/mL). Antibody response peaked at T2 (527.0 [0.4–2500] IU/mL) and declined over T3 (323.0 [0.4–2500] IU/mL) and T4 (141.0 [0.4–2500] IU/mL). Active smokers had lower antibody titers at T2 (*p* = 0.04), T3 (*p* = 0.04), and T4 (*p* < 0.0001) compared with former or never smokers. Peak antibody titers were not associated with any other clinicopathologic characteristic. No significant differences were observed compared with Group B. However, lung cancer patients exhibited significantly decreased antibody titers compared with Group C at T4 (*p* < 0.0001). *Conclusions*: Lung cancer patients demonstrate sufficient antibody response six weeks after the first dose of vaccine against SARS-CoV-2 when vaccinated with two-dose regimens. Rapidly declining antibody titers six weeks after the first dose underline the need for a third dose three months later, in patients with lung cancer, and especially active smokers.

## 1. Introduction

Coronavirus disease 2019 (COVID-19) is an infectious disease caused by severe acute respiratory syndrome coronavirus 2 (SARS-CoV-2) which was firstly identified in Wuhan, China, in December 2019 [1]. A COVID-19 pandemic was declared by WHO in March 2020 [2], and since then, the disease has spread worldwide [1]. Although COVID-19 may present with a variety of symptoms, it predominantly affects the respiratory system, and its severity depends on various risk factors [3].

Active malignancy is considered an independent risk factor for severity and mortality in COVID-19 [4,5,6,7]. Among patients with solid tumors, those with lung cancer have a higher tendency for severe COVID-19 as well as a higher mortality rate (33–42%) [8,9,10]. However, their immune response following vaccination has not been thoroughly studied, with assumptions regarding their immunization status being based on small cohorts [11,12,13,14]. No significant differences in seroconversion rates between patients with thoracic and other type of malignancies are described in these studies [11,12,13,14,15,16,17]. More recently, a large prospective study evaluated SARS-CoV-2 spike (S) antibodies in patients with thoracic malignancies (93.1% with lung cancer) after vaccination [18]. The main finding of this study was that lung cancer patients demonstrated efficient anti-SARS-CoV-2 response after vaccination, especially after the second dose [18]. Moreover, it highlighted the need for a third dose in patients with persistently low antibody titers, especially in the light of emerging SARS-CoV-2 variants [18]. A recent systematic review and meta-analysis of observational studies regarding serologic response in patients with malignancy, described significantly lower antibody response compared with the general population, especially for those with hematologic malignancies. A serologic response was documented in 54% of the patients after the first vaccine dose, which increased to 88% after the second. Results of this review suggest that patients with cancer should continue to follow safety measures including mask-wearing after vaccination and underlines the need for additional strategies for prophylaxis [19].

In lack of large trials assessing the efficacy of vaccination in patients with thoracic malignancies and of universal guidelines for the proper timing of booster doses, crucial questions remain to be answered; namely, (i) which diagnostic tools are the most reliable for evaluation of immune response against SARS-CoV-2, (ii) what is the clinical utility of serologic testing, (iii) what are the antibody titer thresholds that confer protection against severe SARS-CoV-2, (iv) are there any clinicopathologic characteristics that prompt for a weaker immune response following vaccination against SARS-CoV-2, (v) when a third booster dose should be administered.

Here, we sought to prospectively evaluate the humoral response of patients with lung cancer through direct measurement of anti-S antibodies following vaccination against SARS-CoV-2. We assessed whether antibody titers are linked with distinct clinicopathologic characteristics. Finally, we compared the humoral response of patients with lung cancer with different control cohorts, including non-lung cancer patients and healthy controls.

## 2. Materials and Methods

### 2.1. Study Design and Participants

We conducted a prospective non-interventional study involving lung cancer patients followed at the Oncology Unit of 3rd Department of Internal Medicine, “SOTIRIA” General Hospital for Chest Diseases, National and Kapodistrian University of Athens, Athens, Greece. Patients enrolled in the study provided written informed consent. The study was conducted in accordance with the Declaration of Helsinki and approved by the Institutional Review Board (9045/30-3-2021). All three of the following inclusion criteria had to be met for study enrollment: (i) diagnosis of lung cancer, (ii) ongoing anticancer therapy, (iii) vaccination with a two-dose regimen against SARS-CoV-2. Patients with a PCR-confirmed diagnosis of COVID-19 were excluded from the study; patients with elevated antibody titers at baseline (before vaccination) were also excluded. Patients fulfilling the above-mentioned criteria comprised Group A. As is the result in other studies, variation in seroconversion may be observed with respect to cancer type and choice of treatment regimen [19,20]. Thus, a smaller cohort of patients with other types of solid tumors were also enrolled in the study, serving as a control group (Group B). The same exclusion criteria were applied for Group B. Patients were vaccinated according to the national guidelines and the automatic algorithm employed by the Ministry of Health. The research group did not have any role in the vaccine selection or timing of vaccination.

Baseline clinicopathologic characteristics collected included age, gender, body mass index (BMI), smoking status, number of comorbidities associated with severe COVID-19 (including hypertension and/or cardiovascular disease, diabetes mellitus, underlying respiratory disease, chronic kidney disease, and autoimmune disease) [21], type of vaccine, lung cancer histologic subtype, number of previous lines of systemic therapy, type of anticancer therapy, and use of corticosteroids and/or other immunomodulatory agents. Disease progression while on study, evaluated per Response Evaluation Criteria in Solid Tumours (RECIST v.1.1), was also recorded. Serum for antibody measurement was obtained at different time points before and after vaccination; T0: baseline (before vaccination), T1: two to three weeks, T2: six weeks ± one week, T3: 12 weeks ± three weeks, T4: 24 weeks ± three weeks. Patients were followed during the study for possible COVID-19 infection. Those with PCR-confirmed diagnosis of COVID-19 during the study completed the follow up early, in the respective time point before diagnosis. A third group of healthy controls (no cancer diagnosis) were also included in the study (Group C, consisting of healthcare workers) to investigate the differences in seroconversion between lung cancer patients and the general population. Blood samples for antibody titers in Group C were obtained only at T4.

### 2.2. Study End Points

Primary end point of the study was the humoral response (seroconversion) of lung cancer patients after vaccination with one of the available, two-dose vaccine regimens currently approved in Greece (BNT162b2, mRNA-1273, AZD1222).

Secondary end points included the evaluation of time of seroconversion after vaccination, the clinicopathologic parameters associated with seroconversion and the comparison of humoral immune response between lung cancer patients, those with other solid malignancies and with healthy individuals.

### 2.3. Laboratory Analysis

Serum specimens were obtained and quantitative measurement of total antibodies (including IgG) against the receptor binding domain (RBD) of S protein of SARS-CoV-2 was performed (*Roche* Diagnostics, 9115 Hague Rd Indianapolis, IN 46256, USA, Elecsys^®^ Anti-SARS-CoV-2 S, in vitro immunoassay for the quantitative determination of SARS-CoV-2 RBD antibodies). Electrochemiluminescence immunoassay analyzer (*ECLIA*) was used for analysis. The test was performed according to manufacturer’s instructions. Results <0.8 IU/mL were considered negative and >0.8 IU/mL positive. According to the manufacturer’s package insert, sensitivity of the method is 98.8% (95% CI: 98.1–99.3%) and specificity 99.98% (95%CI: 99.7–100.0%) (20). Measurement range was 0.4–250.0 IU/mL. Measurement of specimens with results >250.0 IU/mL was repeated after further dilution (1:10).

### 2.4. Statistical Analysis

Antibody titers between Groups A and B were compared at T0, T1, T2, T3, and T4, whereas antibody titers between Groups A and C were compared only at T4. The t test or one-way analysis of variance was used to compare the means between two or more groups, respectively. The chi-square test was used to compare proportions. All hypothesis testing was performed at a two-sided significance level of α equal to 0.05. Statistical analysis was performed using GraphPad Prism 8 software (GraphPad Software, La Jolla, CA, USA).

## 3. Results

### 3.1. Patient Characteristics

Between 10 January 2021 and 31 December 2021, 125 patients with lung cancer were enrolled in Group A. An additional 35 non-lung cancer patients (including patients with breast, colorectal, pancreatic, renal, head and neck cancer, and ovarian cancer) and 86 healthy individuals were enrolled in Groups B and C, respectively. Three patients were excluded from the analysis due to detectable antibody titers at T0.

Detailed clinicopathologic characteristics of Group A can be seen in Table A1. The median age of the study cohort was 68 years (range 46–91). Ninety-six of the patients were males (76.8%). A 16.0%, 80.0%, and 4.0% of the patients were active, former, and never smokers, respectively. Most of the patients (83.2%) had a diagnosis of non-small cell lung cancer (NSCLC) with the rest (16.8%) having a diagnosis of small cell lung cancer (SCLC). All participants were on systemic anticancer therapy (immunotherapy 48%, chemotherapy 27.2%, combination immunotherapy-chemotherapy 23.2%, targeted therapy with tyrosine kinase inhibitors 1.6%) at the time of enrollment. A 21.6% of the patients experienced radiographic disease progression while on study (according to RECIST, version 1.1). All study participants were vaccinated with a two-dose regimen (80.0% BNT162b2, 8.0% mRNA-1273, 12.0% AZD1222).

Baseline characteristics for Groups B and C can be seen in Table A1.

### 3.2. Evaluation of Humoral Response

All patients included in the analysis had at least two available samples at any of the time points defined per study protocol (T0, T1, T2, T3, T4). Antibody titers for Group A are provided in Table A2. Out of 125 participants, 28 (22.4%) had a measurement at T0, 41 (32.8%) at T1, 89 (71.2%) at T2, 93 (74.4%) at T3, and 73 (58.4%) at T4. Median antibody response was 0.4 IU/mL for T0 (not detectable) and T1 (0.4–48.6). Antibody response peaked at T2 (527.0 [0.4–2500] IU/mL) and declined over T3 (323.0 [0.4–2500] IU/mL) and T4 (141.0 [0.4–2500] IU/mL).

**Figure A1:**
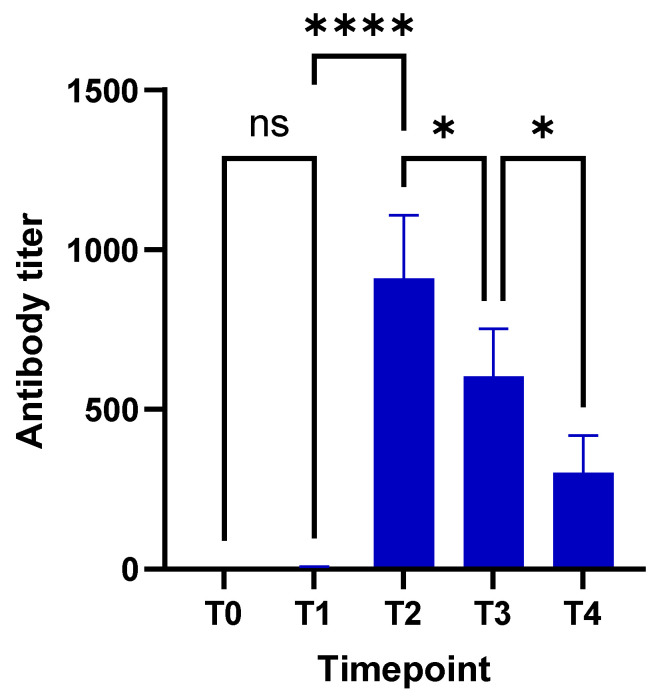
Univariate analysis of seroconversion at different time points for lung cancer patients; mean antibody titers with 95% confidence intervals (y axis) relative to timepoint assessed (x axis); ns: no statistical significance (*p* = 0.99); ****: statistically significant difference (*p* < 0.0001); *: no statistically significant difference between T2 versus T3 (*p* = 0.02) and T3 versus T4 (*p* = 0.03).

Comparison of antibody titers across time in patients with lung cancer (Group A) did not show any significant difference between T0 and T1 (*p* = 0.99; Figure A1). Antibody titers increased significantly between T1 and T2 (*p* < 0.0001) and declined thereafter (T2 versus T3, *p* = 0.02; T3 versus T4, *p* = 0.03).

**Figure A2:**
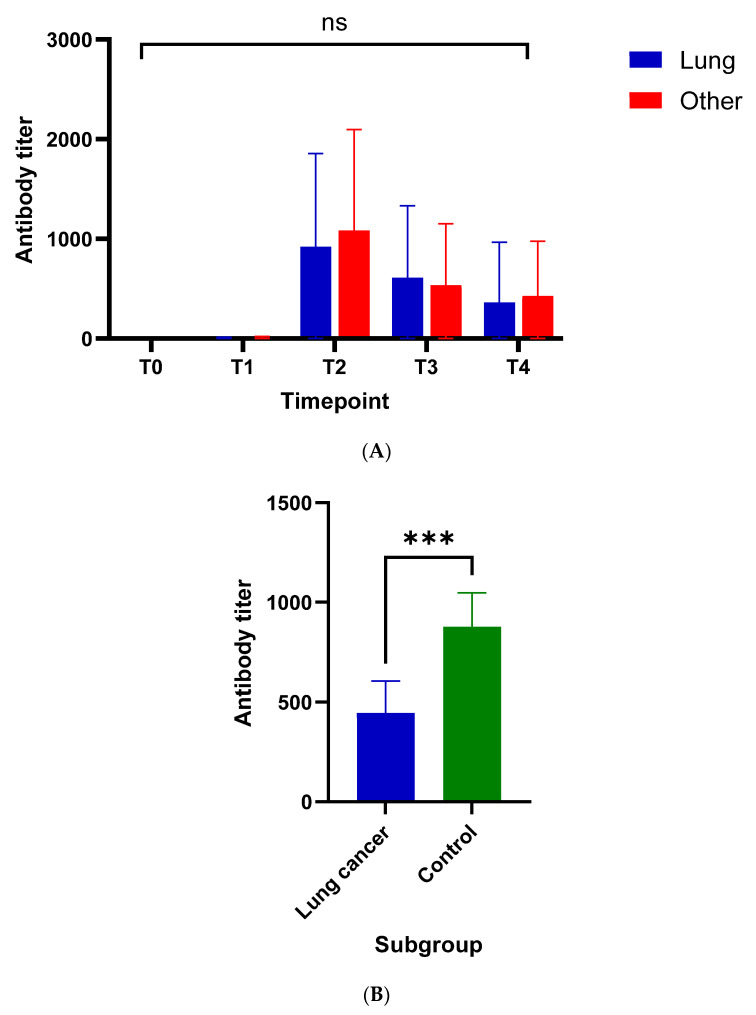
(**A**). Seroconversion comparison between lung and non-lung cancer patients; mean antibody titers with 95% confidence intervals (y axis) relative to timepoint assessed (x axis). (**B**). Seroconversion comparison between lung cancer patients and healthy controls; mean antibody titers with 95% confidence intervals (y axis) relative to timepoint assessed (x axis); ns: no statistical significance; ***: statistically significant difference (*p* = 0.003).

Comparisons of antibody titers between Group A and B did not show any significant differences across time (Figure A2A). However, antibody titers of healthy controls allocated at Group C were significantly higher when compared to Group A at T4 (*p* = 0.003; Figure A2B).

**Figure A3:**
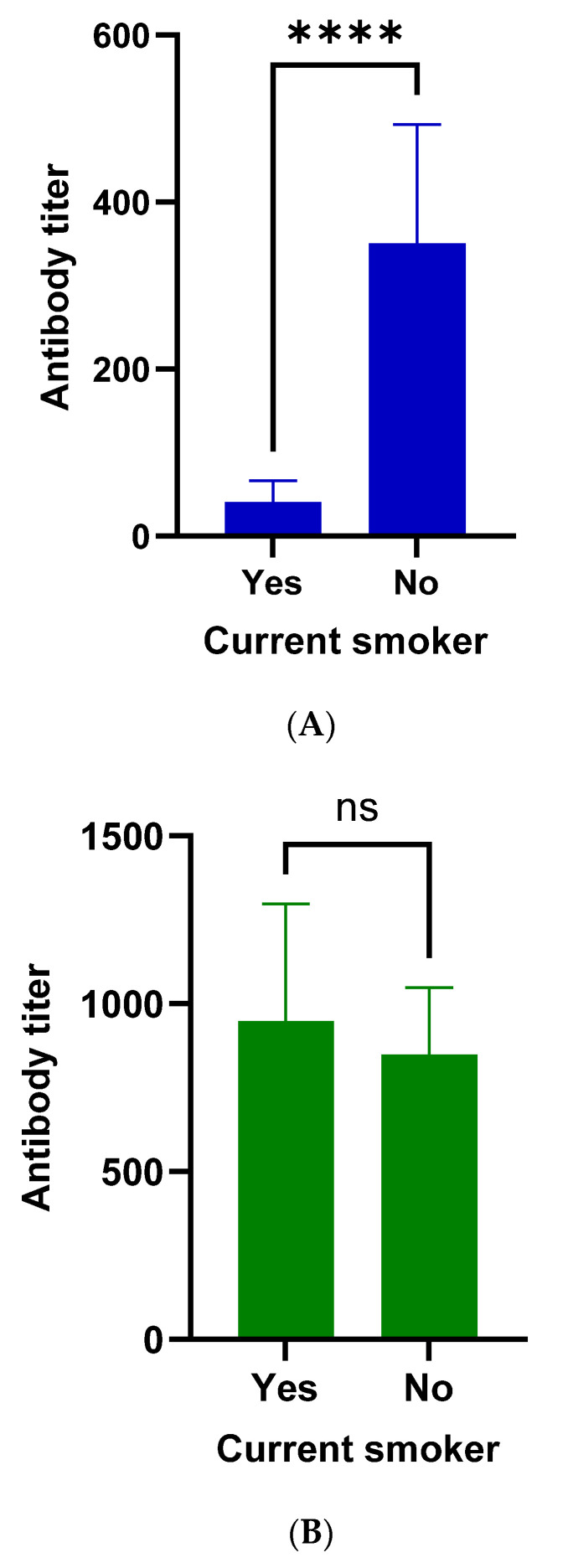
(**A**). Seroconversion comparison between active and former/never-smokers for lung cancer patients; mean antibody titers with 95% confidence intervals (y axis) relative to smoking status (x axis). (**B**). Seroconversion comparison between active and former/never-smokers for healthy controls; mean antibody titers with 95% confidence intervals (y axis) relative to smoking status (x axis); ns: no statistical significance; ****: statistically significant difference (*p* < 0.0001).

Univariate analysis for different clinicopathologic characteristics in Group A showed significantly reduced antibody titers for lung cancer patients who were active smokers compared with lung cancer patients who were former or never smokers at T2 (*p* = 0.04) and T3 (*p* = 0.04), with the difference being more pronounced at T4 (*p* < 0.0001) (Figure A3A). Notably, this was not the case for healthy controls in Group C (*p* = 0.60). No significant differences in seroconversion were observed with respect to other clinicopathologic characteristics.

## 4. Discussion

This study evaluated the immune response of patients with lung cancer through measurement of antibodies against SARS-CoV-2 at different time points after the first dose of vaccination against SARS-CoV-2. The key results of our study include the following: (i) peak seroconversion against SARS-CoV-2 in lung cancer patients is achieved at six weeks after the first vaccine dose, (ii) antibody titers remain high, but gradually decline over a 24-week period after the first vaccine dose, (iii) active smokers demonstrate lower antibody response compared with former or never smokers, (iv) lung cancer patients show lower seroconversion compared with the general population at 24 weeks after the first vaccine dose, (v) no significant differences in seroconversion are observed between lung cancer and non-lung cancer patients.

The results of our study are in concordance with other studies in patients with either thoracic or extra-thoracic malignancies. Although successful seroconversion and T-cell responses are observed in patients with cancer, especially after a second vaccine dose, it is now well documented that the immune response is delayed in such patients [15,16,17]. Moreover, large studies in healthy individuals have demonstrated waning antibody titers over a four-to-six-month period after vaccination, as is the case in patients with cancer. However, healthy individuals, especially young, females, and those without comorbidities, exhibit faster seroconversion and higher antibody titer peaks for the respective time points [22,23]. Our study underlines the above-mentioned findings as this is reflected by differences in seroconversion between lung cancer patients and healthy individuals at 24 weeks after vaccination. In respect of anticancer treatment, most of the studies have not documented any significant deviations in seroconversion among different types of treatment, with the exception of patients treated with B-cell depleting agents [15,16,17]. Use of immunomodulators (including corticosteroids) does not seem to affect immune response, as well. A unique finding of our study is the low antibody response observed in lung cancer patients who are active smokers compared with former or never smokers (Figure A3A). This finding has already been described in the general population [24], however it has not been reported in other studies including cancer patients. Notably, smoking was not associated with seroconversion in healthy controls (Group C) in our study (Figure A3B).

Our study has several limitations. Firstly, evaluation of humoral response does not objectively reflect the immunization status, especially in immunocompromised patients. A variety of serologic tests have been authorized by Food and Drug Administration (FDA). These tests include IgG, IgM, total antibodies, as well as neutralizing antibody assays [25]. Other tests targeting T-cell response, evaluating thus cellular immunity, have also been authorized by FDA [26]. Results from both immunodiagnostic methods are essential for more accurate conclusions concerning immunization status. Secondly, besides the need for assessment of both humoral and cellular immunity, a proper evaluation of immunization status on cancer patients demands large scale epidemiological monitoring for associations between immunodiagnostic results and vaccines’ clinical efficacy (control of clinical utility of the tests). Moreover, adherence to follow up visits at all time points of our protocol was rather challenging in patients with cancer, not only due to the nature of their underlying disease, but also due to COVID-19 restrictions. In addition, regarding comparisons of seroconversion at T4 between lung cancer patients and healthy controls, it has to be underlined that the two groups were not matched and demonstrated major disparities in respect of their clinical characteristics (including age and number of comorbidities). Additionally, baseline measurement (T0) was not obtained in Group C, as most of the individuals were already fully vaccinated against SARS-CoV-2 at the time of enrollment in accordance with the national guidelines for vaccination in healthcare workers.

Despite the mentioned limitations, in our study we used an authorized immunoassay (*Roche* Elecsys^®^ Anti-SARS-CoV-2 S) for evaluation of seroconversion, and we followed a strict time schedule of follow ups. Thus, we believe that our conclusions regarding immune status of our patients against COVID-19, although indirect, are firmly reliable. Moreover, to the best of our knowledge, this is the largest study specifically designed for lung cancer patients with primary endpoint the evaluation of seroconversion and presenting results at predefined time points for a long follow-up period after vaccination.

More studies are needed to replicate our findings. A large ongoing prospective study on lung cancer patients, with a similar follow up protocol with ours, has already announced initial results and a target recruitment accrual of 750 participants is anticipated [27].

## 5. Conclusions

In conclusion, our study highlights the efficacy of vaccination against SARS-CoV-2 in lung cancer patients, especially after a second vaccine dose, and underlines the need for a third booster dose, especially in active smokers, administered as early as three months after the first vaccine dose. Added to current literature, our findings raise the awareness of national health authorities for the need of subsequent booster vaccine doses in patients with lung cancer.

## Data Availability

The data presented in this study are available in the article.

## Appendix A

**Table A1:** Clinicopathologic characteristics of lung cancer patients (Group A).

Characteristics	Group A (N [%])	Group B (N [%])	Group C (N [%])
**Total**	125	35	86
**Gender**			
Males	96 (76.8)	16 (45.7)	24 (27.9)
Females	29 (23.2)	19 (54.3)	62 (72.1)
**Age ***	68 (46–91)	59 (37–85)	50 (21–90)
**BMI ***	26.1 (15.6–49.9)		
**Smoking status**			
Active	20 (16.0)	5 (14.3)	25 (29.1)
Former	100 (80.0)	24 (68.6)	19 (22.1)
Never	5 (4.0)	6 (17.1)	42 (48.8)
**Comorbidities *^,+^**	1 (0–4)	0 (0–3)	0 (0–2)
**Vaccine**			
BNT162b2	100 (80.0)	23 (65.7)	78 (90.7)
mRNA-1273	10 (8.0)	7 (20.0)	3 (3.5)
AZD1222	15 (12.0)	5 (14.3)	5 (5.8)
**Histologic subtype**			
NSCLC	104 (83.2)		
SCLC	21 (16.8)		
**Previous lines of systemic therapy ***	0 (0–5)		
**Treatment regimen**			
Immunotherapy	60 (48.0)		
Chemotherapy	34 (27.2)		
Combination	29 (23.2)		
Targeted therapy	2 (1.6)		
**Radiotherapy**	40 (32.0)		
**Immunomodulatory agents**	27 (26.1)		
**Disease progression**	27 (21.6)		

BMI: Body mass index; NSCLC: Non-small cell lung cancer; SCLC: Small cell lung cancer; *: Range; ^+^: hypertension and/or cardiovascular disease, diabetes mellitus, underlying respiratory disease, chronic kidney disease, and autoimmune disease.

**Table A2:** Seroconversion in lung cancer patients.

Time Points (Weeks)	Number of Patients Sampled (%)	Number of Patients That Received the BNT162b2 Vaccine (%)	Mean/Median IU/mL (Interquartile Range)
**T0 (baseline)**	28 (22.4)	21 (87.5)	0.4/0.4 (0)
**T1 (two to three)**	41 (32.8)	37 (90.2)	5.0/0.4 (3.3)
**T2 (six ± one)**	41 (32.8)	29 (70.7)	910.7/527.0 (1641.5)
**T3 (12 ± three)**	93 (74.4)	69 (74.2)	603.7/323.0 (563.3)
**T4 (24 ± three)**	73 (58.4)	55 (75.3)	301.9/141.0 (312.5)
